# Emotional Eating in Adults: The Role of Sociodemographics, Lifestyle Behaviors, and Self-Regulation—Findings from a U.S. National Study

**DOI:** 10.3390/ijerph18041744

**Published:** 2021-02-11

**Authors:** Roni Elran Barak, Kerem Shuval, Qing Li, Reid Oetjen, Jeffrey Drope, Amy L. Yaroch, Bob M. Fennis, Matthew Harding

**Affiliations:** 1 School of Public Health, Faculty of Social Welfare and Health Sciences, University of Haifa, Haifa 3498838, Israel; relranbar@univ.haifa.ac.il; 2 The Cooper Institute, Dallas, TX 75230, USA; 3 American Cancer Society, Atlanta, GA 30303, USA; Qing.li@cancer.org; 4 Department of Health Management & Informatics, University of Central Florida, Orlando, FL 32816, USA; Reid.Oetjen@ucf.edu; 5 School of Public Health, University of Illinois at Chicago, Chicago, IL 60612, USA; jdrope@uic.edu; 6 Gretchen Swanson Center for Nutrition, Omaha, NE 68114, USA; ayaroch@centerfornutrition.org; 7 Department of Marketing, University of Groningen, 9747AE Groningen, The Netherlands; b.m.fennis@rug.nl; 8 Department of Economics, University of California- Irvine, Irvine, CA 92617, USA; harding1@uci.edu

**Keywords:** emotional eating, lifestyle behaviors, self-regulation, sociodemographics

## Abstract

Background: Emotional eating, the tendency to overeat in response to negative emotions, has been linked to weight gain. However, scant evidence exists examining the prevalence and correlates of emotional eating among large samples of adults in the United States (U.S.). Hence, we examine the relationship among individual and socioeconomic factors, health behaviors, and self-regulation with emotional eating patterns among U.S. adults. Methods: Cross-sectional analysis of 5863 Family Health Habits Survey participants. Multivariable, ordered, logistic regression was employed to examine the relationship between the frequency of the desire to eat when emotionally upset (never, rarely, sometimes, often, and very often) and the independent variables. Results: Analysis reveals that 20.5% of the sample tended to emotionally eat often or very often. Being female, non-Hispanic White, and of younger age were all related to a higher likelihood of emotional eating. Additionally, inability to delay gratification (impatience) was related to an 18% increased likelihood (95% confidence interval (CI) 1.05–1.33) for emotional eating. Finally, emotional eating was significantly related to more frequent fast-food consumption. Conclusions: Program planners might need to develop targeted interventions aimed at enhancing emotional regulation skills while addressing these less healthful behaviors (e.g., fast-food intake) with the goal of obesity and chronic disease prevention.

## 1. Introduction

Obesity is a major public health concern particularly as it leads to increased risk for premature mortality and chronic diseases, including type 2 diabetes, cardiovascular disease, hypertension, stroke, some cancers, as well as soaring healthcare costs [1,2,3,4]. Emotional eating, which refers to the tendency to overeat in response to negative emotions, has been studied extensively over the last decades as a risk factor for obesity and an impediment to weight loss [5,6,7,8,9,10].

Studies have employed various research methods to demonstrate how negative emotions, including sadness, anxiety, stress, or anger, are related to the urge to overeat. For example, laboratory studies indicate that priming a negative affect among obese binge eaters via exposure to a sad film, induces overeating [11], and a meta-analysis of 36 ecological momentary field studies [12], confirmed an increase in negative emotions prior to binge eating episodes. A seminal study by Kaplan and Kaplan, focusing on the psychosomatic interpretation of obesity, posits that eating in response to negative emotions is a learned behavior that aims to diminish the negative state that one is in [13].

Furthermore, research has found a link between emotional eating and weight gain [14] and that enhancing emotional regulation skills should be the focus of interventions aimed at weight loss rather than caloric restriction alone [15]. Hence, it is important to describe the prevalence of emotional eating at a national level, factors predicting it, as well as its corollaries (e.g., associated health-related behaviors), particularly as emotional eating has been linked to adverse health outcomes [10,16]. In this study, we describe emotional eating among a large U.S. sample of adults by individual and socioeconomic factors, health behaviors (e.g., fast-food intake, physical activity), and a key indicator of self-regulatory performance, namely, temporal discounting [17]. Findings help to elucidate factors that are related to emotional eating and might, therefore, inform future intervention programs focused on emotional regulation while eating.

## 2. Materials and Methods

The current study cross-sectionally examines the relationship of sociodemographic factors, lifestyle behaviors, and self-regulation (independent variables) with emotional eating (dependent variable). This is explored using data collected in 2011 from the Family Health Habits Survey (FHHS), which is described elsewhere [18]. Briefly, households from the Nielsen/Information Resources Inc. Consumer Panel were asked to participate in an internet-based survey (i.e., FHHS), which aimed to assess obesity and lifestyle behaviors in families [18]. In the present study, we utilize individual-level data on 5863 adults aged 21 years and above from the FHHS with information pertaining to the independent and dependent variables. The current study received ethics approval from the University of Haifa Institutional Review Board (IRB) as well as exempt status from the Morehouse School of Medicine IRB.

Individual and socioeconomic variables consist of age (21–39, 40–49, 50–59, ≥60 years), race/ethnicity (non-Hispanic White, non-Hispanic Black, Hispanic, other), annual household income (<$30,000, $30,000–44,999, $45,000–69,999, ≥$70,000), household size (continuous), college education (yes/no), marital status (married: yes/no), and self-reported health status (low, medium, high). In addition, body mass index (BMI) was computed using the standard formula (kg/m^2^) based on reported weight and height. BMI was then dichotomized based on obesity (BMI ≥ 30): yes/no [4]. Additionally, participants’ sex was missing for a large proportion (73.9%) of participants [19]. Consequently, a multiple-imputation approach, where the covariates along with participants’ height are considered, was used to impute the missing sex variable [20]. This approach is consistent with a previous FHHS study [19].

The physical activity measure is described elsewhere [21]. Briefly, this measure is adapted from the International Physical Activity Questionnaire (IPAQ) [22], where the metabolic equivalent for task (MET) minutes per week (min/week) are computed based on the frequency, intensity, and duration of the activity [21]. MET min/week were then dichotomized according to the Health and Human Services Physical Activity guidelines (≥500 MET min/week): yes/no [23]. In addition, the frequency of fast-food consumption (eat-in and take out) was based on the reported times per week frequenting these establishments [24]. Participants were also queried regarding the frequency of eating at sit-down restaurants. Both variables were categorized into the following three groups for consistency with previous research: 0–1; 2–3; and ≥4 times per week [19].

We used an established proxy of self-regulatory performance, namely, delay discounting [17,25]. Delay discounting measures assess the ability to exert patience—the extent to which one is willing to forego a smaller, more immediate reward for a larger, but later reward. Thus, delay discounting measures gauge the ability to suppress present-moment impulse in the service of valued longer-term goals with higher patience indicating higher self-regulatory performance [26,27]. In this study, we utilized a survey question on monetary tradeoffs related to delayed discounting. Specifically, participants were asked whether they would prefer to receive $10 in 30 days or larger monetary sums ($12, $15, $18) in 60 days [19]. Based on responses, we calculated delta values, indicative of one’s ability to delay immediate gratification, using the standard exponential discount model [28,29]. As described elsewhere [29], delta values, computed by dividing $10 by the lowest monetary sum one is willing to receive in 60 days, were grouped into three categories: (1) patience (delta = 0.83); (2) medium patience (delta = 0.56 ˅ 0.67); and (3) impatience (delta < 0.56). Whereas patience served as the reference group, the medium patience and impatience categories referred to varying levels of one’s (in)ability to delay gratification.

Participants were asked to state the frequency with which they feel the desire to eat when emotionally upset or stressed. This question was adapted from the emotional eating scale of the Dutch Eating Behavior Questionnaire (DEBQ) [30]. Specifically, participants were asked: “When you are emotionally upset or stressed, how often do you feel the desire to eat?”. They were then asked to choose one of the following verbal expressions of frequency [31]: Never, rarely, sometimes, often, and very often”. Due to its ordinal nature, this variable was entered into ordered logistic regression models as the dependent variable.

The relationship among socioeconomic factors, self-regulation, lifestyle behaviors and emotional eating is examined utilizing two, ordered, logistic regression models. The first model includes socioeconomic variables and self-regulation as independent variables and emotional eating as the dependent variable. The second model adjusts for variables in the first model with the addition of health and lifestyle behavior variables (e.g., obesity, physical activity, frequency of fast-food consumption). In both models, the ordered regression is indicative of the odds of reaching a higher emotional eating score versus remaining in the same score according to the independent variables. Odds ratios (OR) and 95% confidence intervals (CI) were computed. Stata version 15.1 (StataCorp LP, College Station, Texas, USA) was utilized for the analyses, with alpha below 0.05 regarded as statistically significant.

## 3. Results

Participants’ baseline characteristics are described in Table 1. Briefly, 59.2% of individuals were aged 50 years and older, with the largest (81.6%) racial/ethnic group being non-Hispanic White, followed by non-Hispanic Black (7.3%), and Hispanic (5.2%). Less than half (45.6%) were college educated, and 62.7% earned an annual household salary of below $70,000. Regarding participants’ lifestyle variables, 33.6% were obese, 21.5% met physical activity guidelines, and 25.9% frequented fast-food establishments twice a week or more. Moreover, 27.1% were regarded as being impatient; that is, having difficulties in delaying immediate gratification. Finally, 20.5% of participants indicated a tendency for emotional eating often or very often.

**Figure 1:**
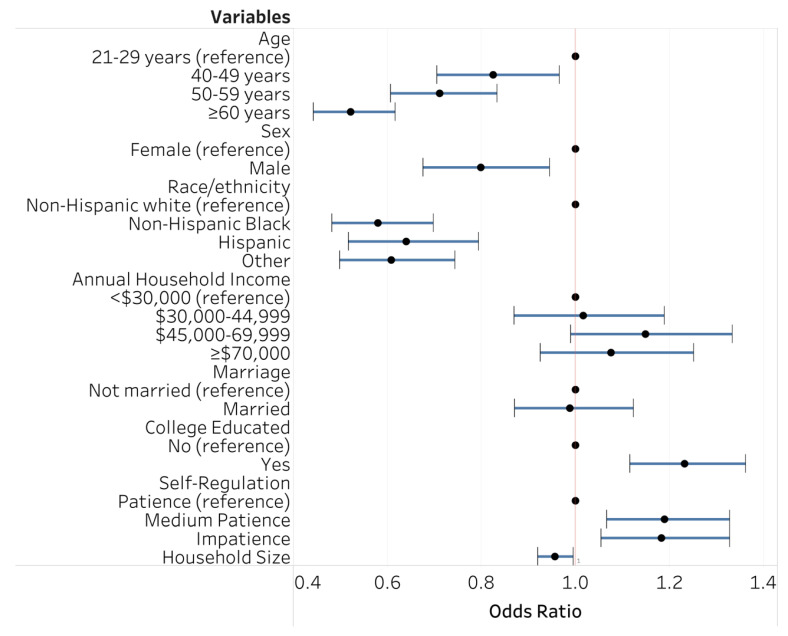
Socioeconomic variables, Self-regulation, and emotional eating ^a^: ordered logistic regression ^b^. ^a^ Emotional eating is based on a survey item pertaining to the desire to eat when emotionally upset or stressed. ^b^ The circles represent odds ratios, and the range bars are the 95% confidence interval. When the horizontal line crosses the vertical line, the relationship is not statistically significant.

Figure 1 depicts the relationship between socioeconomic factors and self-regulation to emotional eating. Analysis reveals that being female, non-Hispanic White, and of younger age were all related to a higher likelihood of emotional eating. For example, non-Hispanic Blacks and Hispanics were less likely (OR = 0.58, 95% CI 0.48–0.70; OR = 0.64, 95% CI 0.52–0.79; respectively) to report higher emotional eating rates than their non-Hispanic White counterparts. Further, having a college education was significantly associated with emotional eating (OR = 1.23; 95% CI 1.12–1.36). Additionally, those who were impatient and had medium levels of patience were 19% (95% CI 1.07–1.33) and 18% (95% CI 1.05–1.33), respectively, more likely to have higher emotional eating scores. Marital status and annual household income, however, were not significantly related to emotional eating.

**Figure 2:**
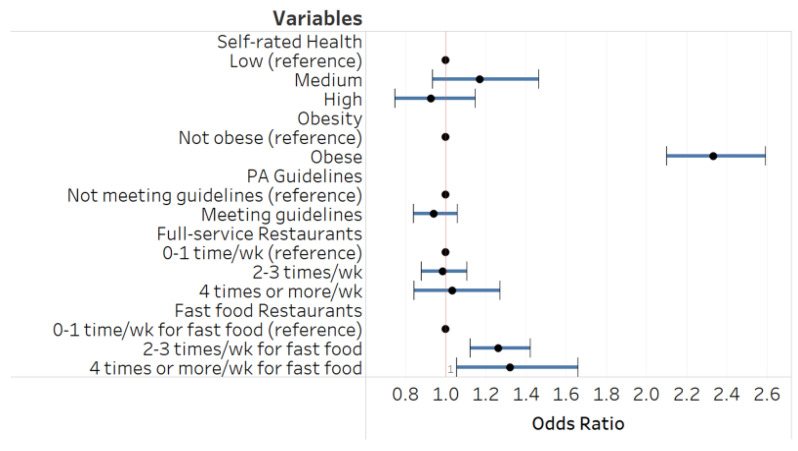
Self-rated health, obesity, lifestyle behaviors, and emotional eating: ordered logistic regression ^a,b^. ^a^ The circles represent odds ratios, and the range bars are the 95% confidence interval. When the horizontal line crosses the vertical line, the relationship is not statistically significant. ^b^ The model adjusts for age, sex, race/ethnicity, marital status, annual household income, education, household size, and self-regulation.

Figure 2 presents the association between lifestyle behavior variables and emotional eating while adjusting for co-variables. Analysis reveals that more frequent fast-food consumption and obesity were each significantly related to emotional eating. For example, those frequenting fast-food establishments 2–3 times a week were ~24% (95% CI 1.10–1.40) more likely to have a higher emotional eating score in comparison to those with a fast-food consumption of 0–1 times weekly (reference group). Full-service restaurant consumption and physical activity as well as self-rated health were not related to emotional eating.

## 4. Discussion

Obesity is a risk factor for chronic diseases and premature mortality [4]. Emotional eating, the tendency to eat in excess when experiencing negative emotions, is related to weight gain and thus obesity risk [14]. Emotional eating also hinders weight loss and weight maintenance [5,6,7,8,9,10]. In the current study, we seek to describe rates of emotional eating among a national sample of adults, while illuminating potential contributing factors to this phenomenon. Findings suggest that approximately one-fifth of adults reported a tendency for emotional eating often or very often, thereby potentially contributing to the obesity epidemic in the U.S. [1,15]. It should be noted that emotional eating was determined via a single survey item assessing the desire to eat when upset or stressed. While it might have been preferable to utilize the complete 13-item emotional-eating subscale of the Dutch Eating Behavior Questionnaire [30], this information was not available in the dataset.

Beyond describing prevalence rates, the present study explores sociodemographic factors related to emotional eating. Specifically, multivariable analysis indicates that younger adults (21–39 years old) were markedly more likely to be emotional eaters. One possible explanation for this finding is that older adults might have a tendency to adhere to routine meal schedules (i.e., breakfast, lunch, dinner) [32], which facilitates meal planning and enhances eating self-efficacy in social situations (e.g., when tempting food is in front of them). Moreover, eating disorders (which are associated with high rates of emotional eating) are more prevalent among younger rather than older adults [33].

Notably, non-Hispanic Blacks and Hispanics reported lower emotional-eating rates than their non-Hispanic White counterparts did. These findings are supported by research suggesting that despite a high prevalence of obesity among African Americans and Hispanics [34], the prevalence of disordered eating behaviors (e.g., emotional eating) among these minority groups is relatively low [35,36,37]. Scant research, however, has specifically examined the underlying mechanisms as to why the prevalence of emotional eating might differ by race/ethnicity. Diggins and colleagues, for example, examined the relationship between stress and emotional eating among African–American female college students [38]. They did not explore, however, how stress might have differentially impacted emotional eating among Whites or Hispanics. It could be plausible that ethnic minorities are more resilient to life stressors [39], and thus less prone to emotional eating in comparison to their ethnic majority counterparts. This supposition, however, warrants future empirical research.

In addition, we examined the relationship between emotional eating and lifestyle behaviors, such as fast-food consumption and physical activity. Study findings indicate that unhealthy lifestyle behaviors (e.g., fast-food consumption) are related to emotional eating while more healthful behaviors (e.g., physical activity) are not. Prior evidence suggests that low distress tolerance (inability to cope with negative emotions) is related to emotional eating [40]. Moreover, the link found between fast food and emotional eating is consistent with previous studies showing that emotional eaters often have a preference for energy-dense foods with abundant saturated fat [41,42]. With regard to physical activity, our findings corroborate a study by Koenders among 1562 U.S. adults observing no significant association between emotional eating and exercise [14]. Thus, while emotional eating and lack of insufficient physical activity are each related to weight gain and maintenance [10,43], they appear not to be directly linked to each other.

Furthermore, current study findings underscore the independent and significant relationship between patience time preferences and emotional eating. That is, those who had difficulties delaying immediate gratification for a larger delayed reward were markedly more likely to eat when emotional than their more patient counterparts were. This finding is consistent with psychological research linking emotional eating behaviors to impulsiveness and self-control [44,45]. These studies, however, measured self-control via a self-report instrument asking participants to rate their ability to resist temptation [46], which might be influenced by conscious or unconscious factors to reinforce self-image [27]. While this approach is widely accepted, eliciting self-control through assigning an objective task, such as in psychological experiments (e.g., crossing out the letter “e” in a text) [47], or multiple list price methodology (in economics) will likely yield a more valid assessment [48]. Hence, in the present study, we utilize the latter approach (i.e., multiple price list methodology), which provides a more robust assessment of self-regulation [49].

The current study has several limitations that should be noted. Its design is cross-sectional, therefore a temporal (and subsequent causal) relationship between the independent variables (e.g., lifestyle behaviors) and dependent variable (emotional eating) cannot be substantiated. Thus, subsequent longitudinal research is needed to establish a cause–effect relationship. Moreover, study variables such as lifestyle behaviors and emotional eating, were self-reported; thus, under or over reporting could have occurred due to social desirability [50]. Nonetheless since standard measures were used to collect information from participants, non-differential misclassification could have occurred which causes a bias of the point estimates toward the null [51]. In addition, the sex variable was missing for a large proportion of the sample; thus, we utilized a multiple-imputation approach to address this limitation. Finally, the data were derived from a U.S. survey that is not nationally representative, and the racial/ethnic minority composition in this sample is lower than that in the U.S. population at large.

## 5. Conclusions

The current study significantly contributes to the literature by determining the prevalence of emotional eating among a national sample of U.S. adults and examining predictive factors of this behavior. Findings reveal that approximately one-fifth of U.S. adults report emotional eating behavior often or very often, and it is more common among younger adults, non-Hispanic Whites, those with a college degree, and with difficulty delaying immediate gratification. Furthermore, an emotional eater might have an increased tendency for obesity and to eat at fast-food establishments more often. Future longitudinal research among large samples is clearly warranted to determine cause–effect relationships. Moreover, as emotional eating is related to obesity and other unhealthy behaviors, program planners might need to develop targeted interventions aimed at addressing these maladaptive health behaviors (e.g., fast-food intake) alongside improving emotional-regulation skills with the goal of obesity prevention and chronic disease prevention.

## Figures and Tables

**Table 1:** Baseline social demographic, lifestyle behaviors, and self-regulation characteristics of study participants ^a,b^.

Characteristic	n	Percentage *
Age (years)		
21–39	786	13.41%
40–49	1605	27.38%
50–59	1796	30.63%
60+	1676	28.59%
Sex		
Male	454	29.67%
Female	1076	70.33%
Race and ethnicity		
Non-Hispanic White	4785	81.61%
Non-Hispanic Black	426	7.27%
Hispanic	308	5.25%
Other	344	5.87%
Annual household income		
<$30,000	1087	18.54%
$30,000–44,999	1030	17.57%
$45,000–69,999	1559	26.59%
≥$70,000	2187	37.30%
Married		
No	4113	70.15%
Yes	1750	29.85%
College Graduate		
No	3187	54.36%
Yes	2676	45.64%
Household Size- Mean (SD)	5863	2.84 (1.43)
Self-rated Health		
Low	331	5.65%
Medium	1356	23.13%
High	4176	71.23%
Self-Regulation		
Patience	2434	41.52%
Medium patience	1837	31.33%
Impatience	1592	27.15%
Obese		
No	3892	66.38%
Yes	1971	33.62%
Meeting PA Guidelines		
No	4603	78.51%
Yes	1260	21.49%
Fast-food restaurants		
0–1 times/week	4344	74.09%
2–3 times/week	1240	21.15%
≥4 times per week	279	4.76%
Full-service Restaurants		
0–1 times/week	4171	71.14%
2–3 times/week	1332	22.72%
≥4 times per week	360	6.14%
Emotional Eating		
Never	1047	17.86%
Rarely	1744	29.75%
Sometimes	1868	31.86%
Often	680	11.60%
Very often	524	8.94%

* If the percentage does not equal 100.0%, this is due to rounding. ^a^ The sex variable is multiply imputed due to missing data; ^b^ PA = physical activity. Meeting PA guidelines here refers to reaching 500 metabolic equivalent task (MET) minutes per week or more to meet the requirements of the Health and Human Services Physical Activity Guidelines for Americans.

## Data Availability

The data used for this study are not publicly available. For data requests, please contact the Nielsen Consumer Panel.
